# Powder X-ray diffraction as a powerful tool to exploit in organic electronics: shedding light on the first *N*,*N*′,*N*′′-tri­alkyl­diindolocarbazole

**DOI:** 10.1107/S2052520622001858

**Published:** 2022-03-29

**Authors:** Anna Vilche, Roger Bujaldón, Xavier Alcobé, Dolores Velasco, Cristina Puigjaner

**Affiliations:** a X-ray Diffraction Unit, Scientific and Technological Centers, University of Barcelona, Lluís Solé i Sabarís 1-3, 08028 Barcelona, Spain; b Grup de Materials Orgànics, Institut de Nanociència i Nanotecnologia (IN^2^UB), Departament de Química Inorgànica i Orgànica, Secció de Química Orgànica, Universitat de Barcelona, Martí i Franquès, 1, 08028 Barcelona, Spain

**Keywords:** organic semiconductor, OTFT, crystal structure determination, powder X-ray diffraction, SDPD, TOPAS, diindolocarbazole

## Abstract

The first crystal structure of a fully *N*-alkyl­ated diindolocarbazole derivative, namely, 5,8,14-tri­butyl­diindolo[3,2-*b*;2′,3′-*h*]carbazole (**1**, C_36_H_39_N_3_), has been determined from laboratory powder X-ray diffraction (PXRD) data. A com­plex trigonal structure with a high-volume unit cell of 12987 Å^3^ was found, with a very long *a*(=*b*) [52.8790 (14) Å] and a very short *c* [5.36308 (13) Å] unit-cell parameter (hexa­gonal setting). The detailed analysis of the inter­molecular inter­actions ob­served in the crystal structure of **1** highlights its potential towards the imple­mentation of this core as a semiconductor in organic thin-film transistor (OTFT) devices. Since the mol­ecule has a flat con­fig­uration reflecting its π-conjugated system, neighbouring mol­ecules are found to stack atop each other in a slipped parallel fashion *via* π–π stacking inter­actions between planes of *ca* 3.30 Å, with a centroid–centroid distance between the aromatic rings corresponding to the shortest axis of the unit cell (*i.e.* *c*). The alkyl­ation of the three N atoms proves to be a decisive feature since it favours the presence of C—H⋯π inter­actions in all directions, which strengthens the crystal packing. As a whole, PXRD proves to be a valuable option for the resolution of otherwise inaccessible organic crystal structures of inter­est in different areas.

## Abstract 2

The crystal structure determination of *N*,*N*′,*N*′′-tri­butyl­diindolocarbazole from laboratory X-ray powder diffraction data is fully described. The analysis of the aromatic inter­molecular inter­actions allows the com­prehension of this π-conjugated system, which may have great potential with respect to organic electronics.

## 1. Introduction

Organic semiconductors have become, in the optoelectronic field, potential alternatives to the conventional inorganic materials. Some of the enticing advantages associated with them are the easy modulation of their properties, their lower cost and the feasibility to fabricate large-area, lightweight and flexible displays (Bronstein *et al.*, 2020 ▸; Yao *et al.*, 2016 ▸). In fact, their applicability in devices such as organic thin-film tran­sistors (OTFTs) and organic light-emitting diodes (OLEDs) have been extensively demonstrated (Wang *et al.*, 2017 ▸; Huang *et al.*, 2019 ▸). Nevertheless, the demand for im­proved materials, especially in terms of charge transport and air stability, dictates further investigation.

The inter­molecular disposition of the semiconductor within the active layer is crucial in determining the performance of the device, but this can be challenging to predict beforehand in organic structures. Indeed, an appropriate arrangement that facilitates an effective charge transport between neighbouring mol­ecules is highly desired (Campbell *et al.*, 2017 ▸). Thus, an exhaustive analysis of the crystal structure improves the com­prehension of such a process and paves the way towards subsequent structural optimizations or new enhanced designs.

**Figure 1:**
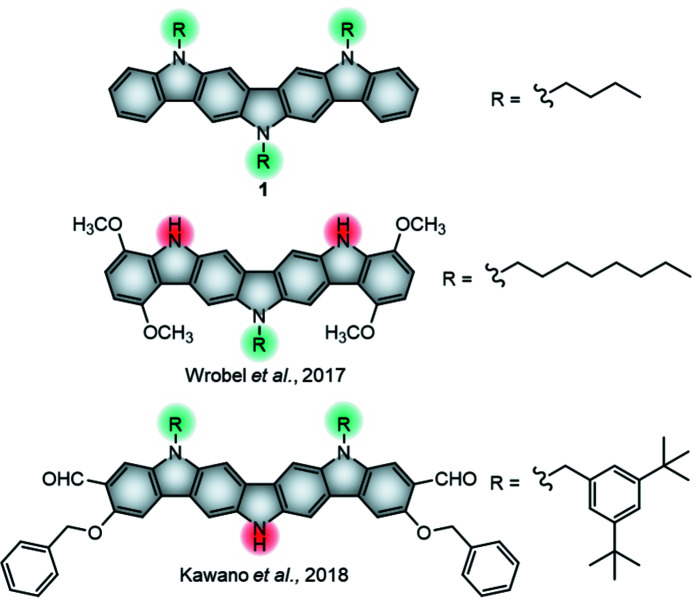
The chemical structures of the elucidated diindolocarbazole derivative **1** and the two derivatives reported previously in the literature. The grey region delimitates the diindolocarbazole core, whereas the colour of the N atoms indicates whether they are alkyl­ated (green) or unprotected (red).

Ladder-type mol­ecules, which feature large and planar aromatic cores, are particularly desirable for high-performing OTFTs because of their ease of establishing inter­molecular π–π stacking inter­actions (Chen *et al.*, 2018 ▸; Cai *et al.*, 2018 ▸; Bujaldón *et al.*, 2021 ▸). A prime example of this type of material is the diindolo[3,2-*b*:2′,3′-*h*]carbazole core. In fact, similar structures, like indolo[3,2-*b*]carbazole and tri­indole, have excelled as air-stable semiconductors in OTFTs as a result of their advantageous inter­molecular dispositions (Reig *et al.*, 2015 ▸). The diindolocarbazole core features an ionization potential of ∼5.1 eV that closely fits the Au work function, and an electron affinity of ∼2.5 eV (Srour *et al.*, 2016 ▸). As reported, this preliminary data agrees with the possibility of a suitable hole injection and transport while also anti­cipating considerable long-term stability. However, the properties of this core integrated in optoelectronic devices remain unexplored. In terms of crystallographic studies, only two derivatives have been reported to date (Kawano *et al.*, 2018 ▸; Wrobel *et al.*, 2017 ▸), which were elucidated *via* conventional single-crystal X-ray diffraction (SXRD). Unfortunately, the aforementioned examples possess one or more unprotected N atoms, which is typically unfavourable in OTFTs (Reig *et al.*, 2018 ▸). The herein studied diindolocarbazole derivative, **1**, on the other hand, possesses a promising homogeneous alkyl­ation pattern with three butyl chains. Fig. 1 ▸ displays its chemical structure, as well as those of the two com­pounds already published, for com­parison. It should be mentioned also that the two previously described crystal structures incorporate different solvent mol­ecules into the packing as a result of the formation of the respective single crystals. This is inconvenient, since final devices usually require the absence of solvent within their active layers to function properly. Consequently, the acquisition of solvent-free crystal structures is crucial to compile realistic and unbiased information about the intermolecular disposition of the material for subsequent optoelectronic applications. The elucidation of the crystal structure of **1** was therefore sought-after information in moving towards the implementation of this core in OTFT devices.

SXRD is the most widely used method for the determination of crystal structures due to the high success of this approach. In spite of numerous attempts, however, our efforts to produce single crystals of **1** suitable for analysis were fruitless. The use of a polycrystalline material for the diffraction experiment was consequently inevitable. Structure determination from powder diffraction (SDPD), while by no means routine, has emerged as a realistic option for determining crystal structures and has become an active research area (Meden *et al.*, 2015 ▸). Besides, elucidation through this procedure does not involve the crystallization of the material in a solvent as opposed to SXRD. Hence, the resulting crystal structure is less prone to embed solvent molecules, which is a mandatory factor in this study.

Although single-crystal and powder diffraction patterns contain essentially the same information, the diffraction data are distributed in three-dimensional (3D) space or com­pressed into one dimension, respectively. As a consequence, there is usually considerable overlap of the peaks in the powder diffraction pattern, resulting in a loss of information. Whereas reflections are well resolved and can be measured individually in the single-crystal case, they can overlap partially or com­pletely in the observed powder diffraction pattern. The problem increases with the size of the unit cell, in the absence of strong scatterers and is generally greater when the symmetry is lower. In recent years, developments in high-resolution PXRD instrumentation, as well as com­putational resources and well-developed algorithms, have reached a level where SDPD has become a powerful tool for structural characterization (Černý, 2017 ▸).

**Figure 2:**
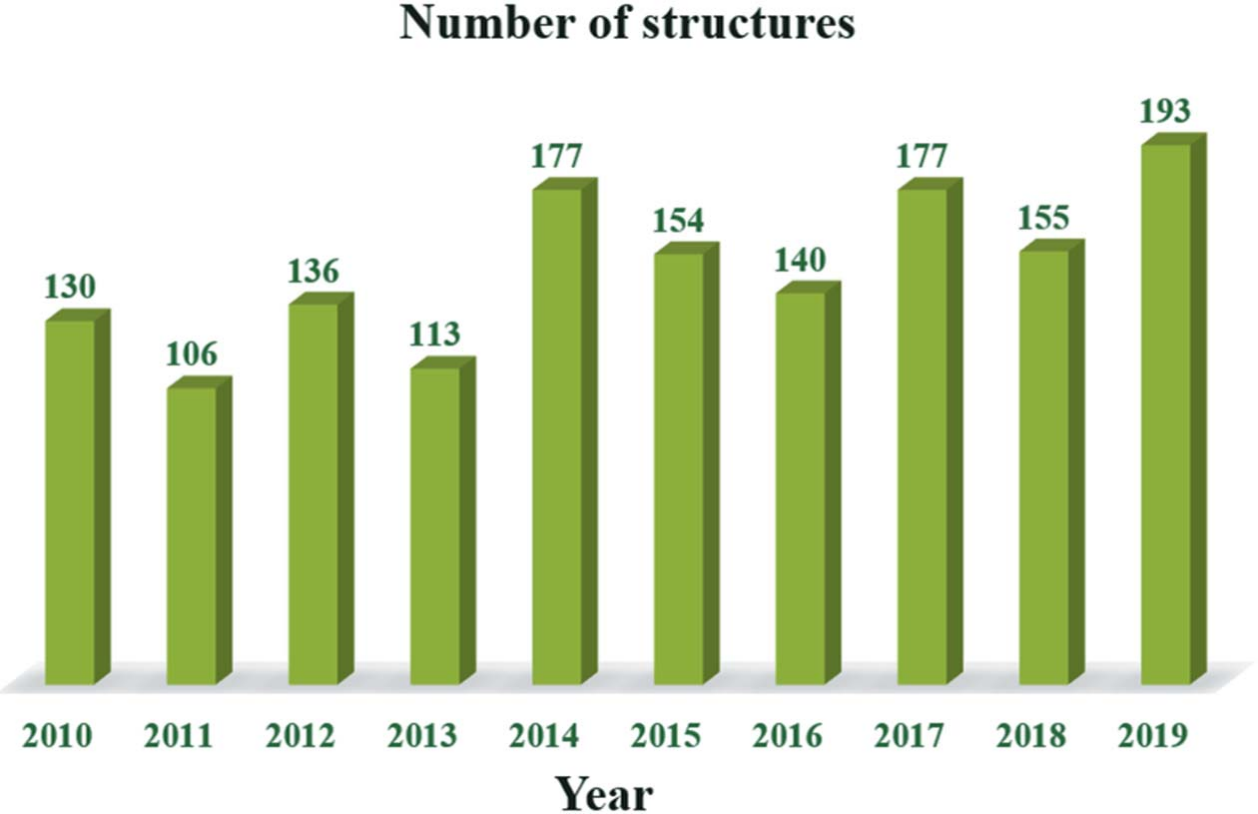
The number of organic crystal structures determined from PXRD data deposited in the CSD during the last decade.

Indeed, the number of organic structures solved annually from PXRD data has increased during the last decade. Fig. 2 ▸ shows the number of structures determined from PXRD data deposited in the Cambridge Structural Database (CSD; Groom *et al.*, 2016 ▸) from 2010 to 2019. The criteria used in this search are the following: 3D coordinates determined, any *R* factor, not disordered, no errors and only organics. Although there is a growing number of crystal structures solved using PXRD, they represent less than 1% of the structures deposited in the CSD. Regarding the application of PXRD in organic electronics, the elucidation of the well-established asymmetrically-alkylated C_8_-BTBT ([1]benzothieno[3,2-*b*]benzo­thio­phene) by Gbabode *et al.* (2014 ▸) and the recent advances from Ishii *et al.* (2020 ▸) towards a more straightforward approach to evaluate organic semiconductors are prime examples of its usefulness in this topic. SDPD, far from being automated, is an active research area where different software has been developed.

Therefore, in the present study, we explore the crystal structure determination of **1** from PXRD data and analyse its inter­actions in order to gain new insight into this core as a promising semiconductor for OTFT devices.

## 2. Experimental

### 2.1. Sample preparation

Compound **1** was synthesized from 2,7-di­bromo-9*H*-carbazole following a reported method (Srour *et al.*, 2016 ▸). Characterization data of **1**, ^1^H NMR (500 MHz, C_6_D_6_): δ (ppm) 8.32 (*d*, *J* = 7.8 Hz, 2H), 8.31 (*s*, 2H), 8.17 (*s*, 2H), 7.53 (*ddd*, *J* = 8.2, *J* = 7.1, *J* = 1.2 Hz, 2H), 7.34 (*m*, 2H), 7.30 (*d*, *J* = 8.1 Hz, 2H), 4.21 (*t*, *J* = 7.1 Hz, 2H), 4.07 (*t*, *J* = 7.1 Hz, 4H), 1.78 (*m*, 2H), 1.66 (*m*, 4H), 1.34–1.10 (*m*, 6H), 0.76 (*t*, *J* = 7.4 Hz, 3H), 0.70 (*t*, *J* = 7.3 Hz, 6H). ^13^C NMR (125 MHz, C_6_D_6_): δ (ppm) 142.4, 138.2, 136.3, 126.0, 123.8, 123.8, 123.7, 120.8, 118.5, 108.9, 99.3, 99.2, 43.5, 43.2, 31.3, 31.1, 21.0, 20.9, 14.1, 14.0. HRMS (ESI–MS) (*m*/*z*): calculated for C_36_H_39_N_3_ *M* ^+**.**^, 513.3138; found 513.3139. The subsequent PXRD measurements were carried out by introducing some of the powder material in a 0.5 mm diameter Hilgenberg glass capillary.

### 2.2. Instrument and experimental conditions

PXRD patterns were obtained on a PANalytical X’Pert PRO MPD diffractometer of radius 240 mm in a transmission con­fig­uration with a spinner glass capillary sample holder, using Cu *K*_α1+2_ radiation (λ = 1.5418 Å) with a focalizing elliptic mirror and a PIXcel detector working at a maximum detector active length of 3.347 Å. Incident and diffracted beam 0.01 radians soller slits and incident beam slits defining a beam height of 0.4 mm were used with the sample placed in a glass capillary. 22 consecutive 2θ scans were measured and added from 2 to 70° in 2θ, with a step size of 0.013° and a measuring time of 700 s per step (total measuring time 88 h).

### 2.3. Structure determination from the powder X-ray diffraction approach

**Figure 3:**
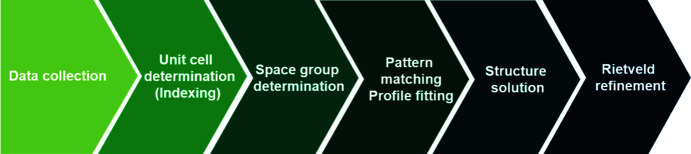
Structure determination from the powder X-ray diffraction (PXRD) procedure.

The SDPD procedure, irrespective of the software used, always consists of the steps shown in Fig. 3 ▸ (David & Shankland, 2008 ▸).

For a high quality data collection it is vital to ensure that the experimental conditions yield angular accuracy and a proper choice of slits, control over the morphology and the size of the sample, and the choice of transmission instead of reflection geometry avoid preferential orientation problems (Bergese *et al.*, 2001 ▸).

The indexing process, *i.e.* analysis of the peak positions in the powder diffraction pattern in order to detect the correct unit-cell parameters and crystal system, is still a bottleneck, especially in cases of low-resolution powder patterns or for triclinic symmetry. Some of the programs used include *DICVOL*, *TREOR* (Werner *et al.*, 1985 ▸), *ITO* (Visser, 1969 ▸), *CRYSFIRE* (Shirley, 2004 ▸) and *DAJUST* (Vallcorba *et al.*, 2012 ▸). Our personal choice is the dichotomy algorithm, nicely implemented in the program *DICVOL04* (Boultif & Louër, 2004 ▸). This allows automatic searching for cubic to monoclinic systems with the volume range estimated. The indexing of triclinic systems is also possible but more difficult. The final results are listed using the standard M20 and F20 figures of merit.

Firstly, the powder X-ray diffractogram of **1** was in principle well indexed to a monoclinic cell with the unit-cell parameters *a* = 5.36, *b* = 26.40, *c* = 15.35 Å, β = 96.6° and *V* = 2156 Å^3^. Taking into account the unit-cell volume, the mol­ecular weight of **1** and an estimated density value of 1.2 Mg m^−3^, the number of mol­ecules in the unit cell was calculated to be *Z* = 3.

Indexing is followed by space-group determination, which is based on systematic extinction analysis. In the present case, the unit cell was primitive and a binary helicoidal axis was discarded due to the presence of the 010 reflection. Moreover, *a*-, *c*- and *n*-glide planes were also discarded as reflections such as 100, 001 and 20 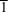 were present. Therefore, the possible space groups for which structure solution should be attempted were *P*2, *Pm* and *P*2/*m*. Considering the multiplicity of these space groups, 1.5 would be the number of mol­ecules in the asymmetric unit for *P*2 and *Pm*, and 0.75 for *P*2/*m*.

At this stage, we recognized the possibility that this monoclinic cell could be a subcell of a higher symmetry and larger-volume cell. Thus, we went back to the indexing step and increased the maximum unit-cell parameters to 60 Å and the maximum volume to 15000 Å^3^. The PXRD diffractogram was perfectly indexed to a hexa­gonal cell with the unit-cell parameters *a* = 52.80, *c* = 5.36 Å and *V* = 12933 Å^3^. In fact, the largest parameter of this higher-symmetry cell was twice the largest parameter of the monoclinic cell and the shortest unit-cell parameters are the same. Now, given the new volume, the unit-cell content was set to 18 mol­ecules. On the basis of systematic absences, a rhombohedral *R* lattice fitted perfectly but the space group could not be assigned unambiguously since in principle all the possible trigonal groups, *i.e. R*3, *R* 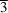 , *R*32, *R*3*m*, *R*3*c*, *R* 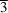 *m* and *R* 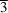 *c*, were com­patible with the observed reflections.

**Figure 4:**
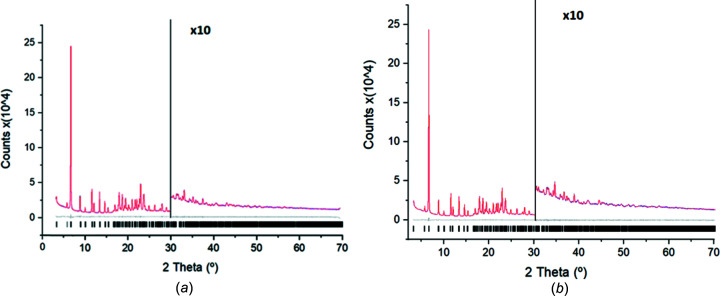
Pattern matching Pawley fit plots for the (*a*) monoclinic and (*b*) trigonal cells; agreement factors: *R*_wp_ = 1.87% and *R*_wp_ = 1.53%, respectively. Both plots show the experimental PXRD profile (blue solid line), the calculated PXRD profile (red solid line) and the difference profile (grey, lower line). Black tick marks indicate the peak positions.

The aim of the next step, namely, so-called pattern matching or profile fitting, is to fit the com­plete experimental PXRD pattern by refinement of variables. These variables describe the peak positions (unit-cell parameters and zero-point shift parameter), the background intensity distribution and the peak intensities, widths and shapes. No structural model is used. The goal is to obtain reliable values of the variables that describe different features of the powder diffraction pattern in preparation for the subsequent stages of the structure determination process. Traditional approaches use peak intensities but many direct-space methods are based on com­parison of the com­plete powder diffraction pattern using a whole profile figure of merit, such as *R*_wp_, and, in this case, the intensity data extracted from the powder diffraction pattern are not used. The two most common applied techniques for pattern matching are those developed by Pawley and Le Bail. In the present case, Pawley (1981 ▸) refinement by means of the *TOPAS* software (Version 6; Coelho *et al.*, 2011 ▸; Coelho, 2018 ▸) was performed. The background was modelled with a 20th-order Chebyschev polynomial and a 1/*X* background function was applied. The instrumental contribution to the diffraction profile was calculated with the Fundamental Parameters Approach (Cheary *et al.*, 2004 ▸). The peak width was modelled with the double-Voigt approach by considering only the Lorentzian contribution of the crystallite size and only the Gaussian contribution to the microstrain. Moreover, the Stephens (1999 ▸) model for anisotropic line broadening was applied. Fig. 4 ▸ shows a com­parison of the Pawley fits for the monoclinic [Fig. 4 ▸(*a*)] and for the trigonal [Fig. 4 ▸(*b*)] cells found in the previous indexing process. Both pattern matchings fit very well the com­plete experimental PXRD pattern, with the agreement factor for the trigonal cell being slightly better (1.53%) than the *R*_wp_ value for the monoclinic cell (1.87%).

Structure solution procedures can be divided into two groups: reciprocal space methods and direct space methods (Černý & Favre-Nicolin, 2007 ▸). On the one hand, the traditionally used reciprocal space methods involve intensity extraction algorithms working on the reciprocal space. On the other hand, the so-called direct space methods or global optimization are based on pattern modelling algorithms working on the direct space and using the chemical knowledge from that space. Hybrid methods iterating between both spaces are known as well.

Nowadays, the most popular procedure to solve organic crystal structures from powder diffraction data is the direct space (also called real space) method. The space group, unit-cell parameters and an initial mol­ecular geometry of the com­ponents in the asymmetric unit are given as input. Starting from a random arrangement of mol­ecules, trial structures are generated in direct space by translating and rotating the mol­ecules within the unit cell, and the suitability of each trial structure is assessed by direct com­parison between the calculated and observed diffraction patterns (Harris *et al.*, 2001 ▸). Intra­molecular degrees of freedom (DOF), such as rotations around single bonds, are also adjusted. Several global optimization techniques are used in direct space SDPD, such as Monte Carlo/Simulated Annealing or Parallel Tempering and Genetic Algorithm techniques (Černý, 2017 ▸). In powder diffraction, the most often used cost function is the weighted profile *R*_wp_ factor calculated over the whole powder pattern.

**Figure 5:**
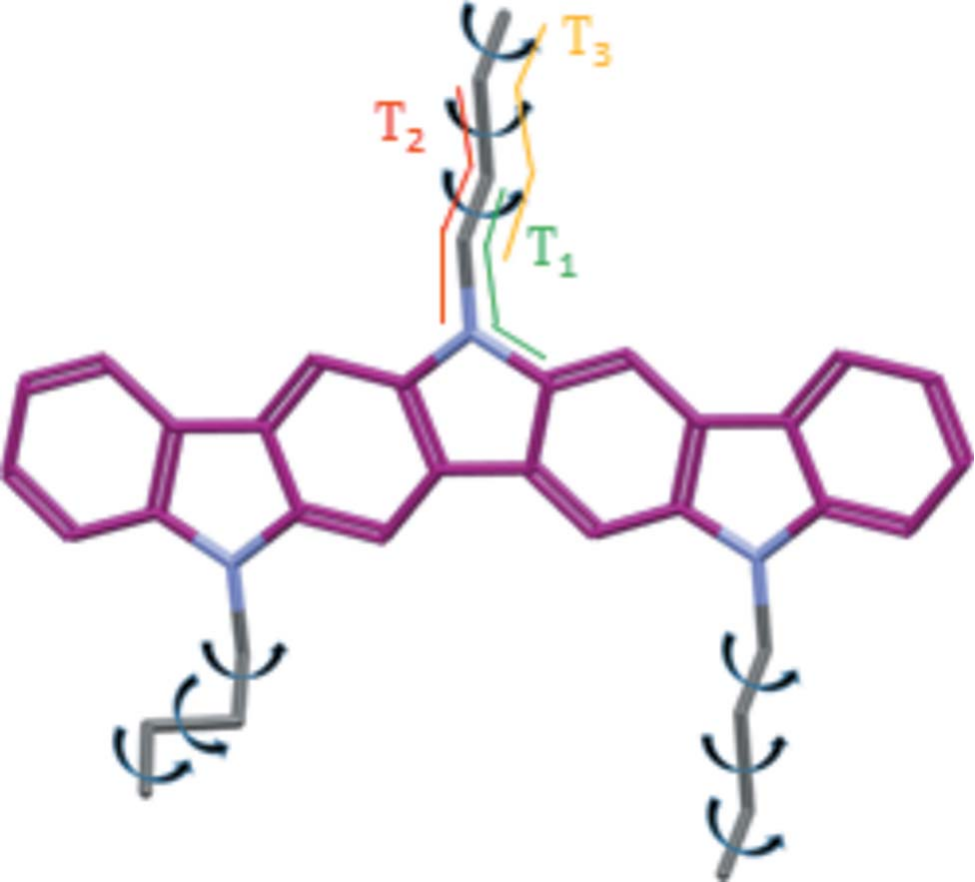
Starting model of **1** used in the Simulated Annealing solution process in *TOPAS*. The torsion angles refined are indicated by arrows and three of them are specified in one of the alkyl chains.

In the present case, the solution of the crystal structure of **1** was attempted using the direct space methodology for each of the possible space groups. The starting model for the crystal structure determination was optimized previously with the program *SPARTAN* (Young, 2001 ▸) and some constraints were introduced, considering the mol­ecule as a rigid body using the *Z*-matrix notation, which was allowed to rotate and translate in the three directions (3 orientational + 3 positional DOFs) within the cell. Planarity restraints were applied to the aromatic rings and nine torsion angles of the flexible aliphatic chains were refined as is shown in Fig. 5 ▸. H atoms were not included in the model at this stage.

The crystal structure was solved using the Global Optimization Simulated Annealing approach integrated in *TOPAS*. The best fit to the experimental PXRD data was found for the space group *R* 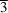 ; this solution was also chemically sensible (Table 1 ▸).

The crystal structure was subsequently refined by the Rietveld (1969 ▸) method, also by means of *TOPAS* software, giving satisfying results with low *R*_wp_ values (Toby, 2006 ▸). In this final step, the variables that define both the structural model and the powder diffraction profile are adjusted by least-squares methods in order to obtain the optimal fit between the experimental and calculated powder X-ray diffractograms. H atoms attached to C atoms were placed at calculated positions.

Several attempts were made to refine the isotropic displacement parameters. Two different *B*_eq_ values were defined, one for the 27 core non-H atoms plus three attached to core C atoms, and another for the remaining nine alkyl C atoms. After refinement of these parameters, either by leaving them free or by setting them to specific values, worse *R*_wp_ and *R*_Bragg_ values were always obtained. Finally, *B*_eq_ values were set to 3 Å^2^ for C and N, and 10 Å^2^ for H atoms, to obtain the lowest *R*_wp_ value, with a meaningful *B*_eq_ value (*i.e.* not a negative value). Furthermore, a preferred orientation correction, the eighth-order spherical harmonics function (Whitfield, 2009 ▸), was applied, as implemented in the *TOPAS* software.

**Figure 6:**
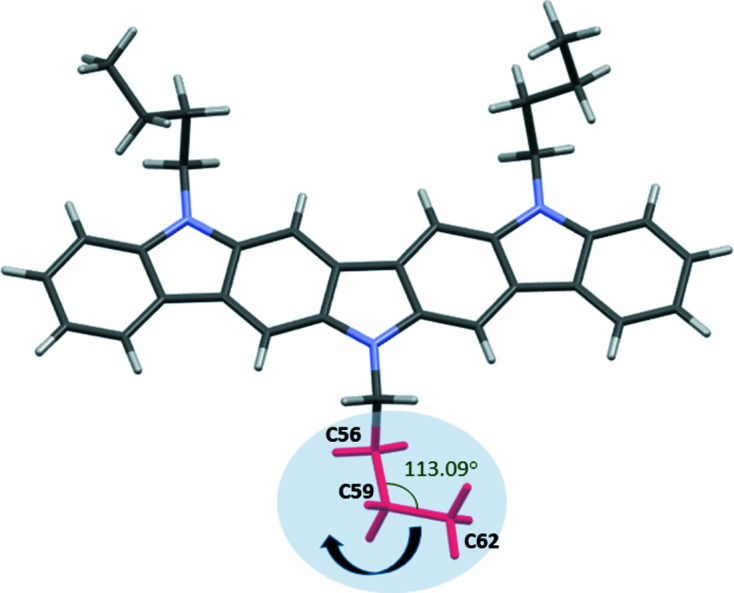
The crystal structure determined for **1** after initial Rietveld refinement but before angle refinement. The labelled atoms indicate the angle that should be modified.

During the Rietveld process, the *checkCIF* report (Spek, 2020 ▸; https://checkcif.iucr.org/) was used to examine the consistency and integrity of the proposed crystal structure. Some inter­molecular inter­actions involving the central alkyl chain were determined to be too short, specifically those labelled in Fig. 6 ▸. Thus, a feasible correction was to rotate the angle involving the three terminal C atoms of the alkyl chain.

**Figure 7:**
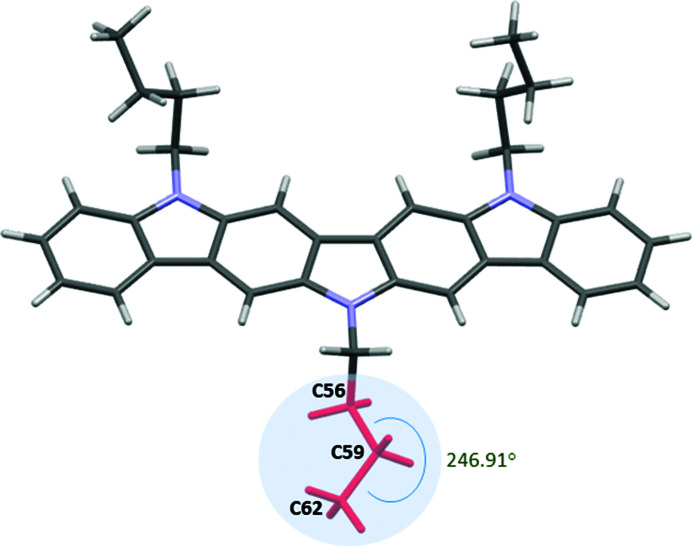
The crystal structure of **1** after angle rotation during Rietveld refinement.

Therefore, the opposite angle involving C56—C59—C62 was calculated by subtracting its initial value from 360°, giving a value of 246.91°, which was introduced in the corresponding *Z*-matrix used to define the rigid body. Fig. 7 ▸ shows the new conformation obtained after the angle rotation which eliminates the *checkCIF* alerts derived from the exceedingly short inter­molecular contacts.

## 3. Results and discussion

**Figure 8:**
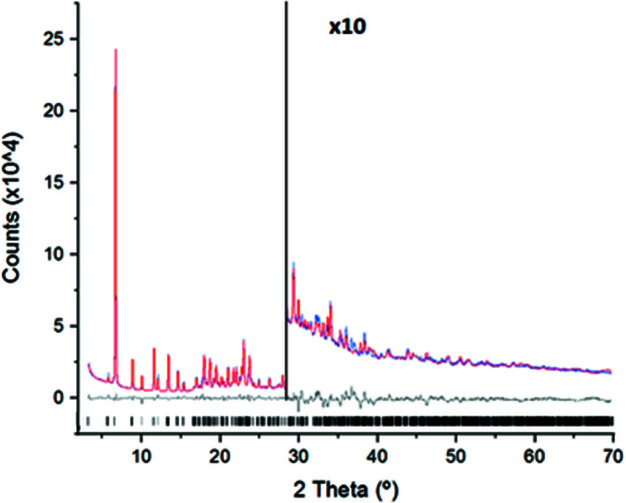
(*a*) Final Rietveld plot for the crystal structure refinement of **1**; agreement factors: *R*_wp_ = 4.82% and *R*_p_ = 3.54%. The plot shows the experimental PXRD profile (blue solid line), the calculated PXRD profile (red solid line) and the difference profile (grey, lower line). Black tick marks indicate the peak positions.

Compound **1** crystallizes in the trigonal space group *R* 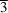 with a high-volume unit cell of 12987.1 (8) Å^3^ and 18 mol­ecules inside it. The final Rietveld plot of the experimental and calculated data is shown in Fig. 8 ▸. The *TOPAS* Rietveld refinement gives an *R*_wp_ value of 4.82% that matches very favourably with the Pawley *R*_wp_ value of 1.53%. Table 2 ▸ summarizes the most relevant parameters of the crystal structure determination and refinement of **1**.

**Figure 9:**
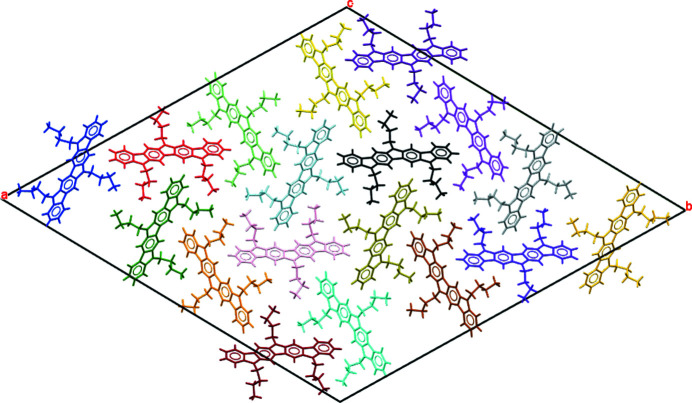
Packing of **1** along the *c* axis showing the 18 mol­ecules in the unit cell.

**Figure 10:**
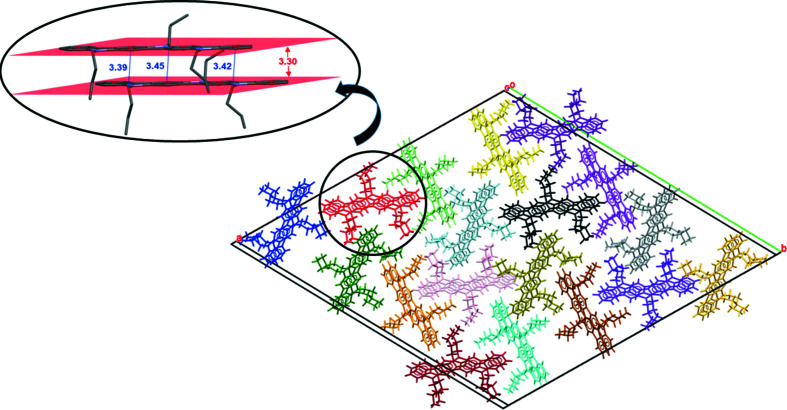
Strong π–π parallel-displaced stackings (shortest inter­molecular π–π distance = 3.30 Å) and C—H⋯π inter­actions (C⋯centroid distances in blue: 3.39, 3.42 and 3.45 Å) between parallel mol­ecules of **1** along the *c* axis.

Fig. 9 ▸ shows the packing of the crystal structure of com­pound **1**. Compound **1** lacks hydrogen-bond donor and acceptor groups. Instead, its main backbone consists of an aromatic system com­posed by four benzene rings fused with three inter­calated pyrrole heterocycles. Thus, the stabilizing impact of the aromatic inter­actions on this structure is expected to be maximal, and certainly structure-directing. Fig. 10 ▸ shows the π–π and C—H⋯π inter­actions established between the aro­matic systems of contiguous mol­ecules along the *c* axis. Since the mol­ecule has a flat con­fig­uration derived from its π-con­jugated system, neighbouring mol­ecules assemble atop each other in a parallel-displaced fashion *via* π–π stacking inter­actions. Indeed, the shortest π–π stacking distance found corresponds to 3.30 Å, which should grant an adequate overlap and charge transport along the material layers. The centroid–centroid distance between molecules of 5.36 Å corresponds to the length of the *c* axis. The angle between the planes of the aromatic rings of 0° and the lateral offset relative to one another of 4.23 Å leads to a favourable offset π-stacked geometry as electronic repulsion dominates in a face-to-face π-stacked geometry (Hunter & Sanders, 1990 ▸). Considering the packing motifs typically seen in polycyclic aromatic hydro­carbons (Desiraju & Gavezzotti, 1989 ▸), the arrangement of **1** can be related to a γ type, which is mainly governed by aromatic C⋯C (and C⋯N in this case) inter­actions between parallel-translated mol­ecules. On the other hand, materials such as penta­cene (Campbell *et al.*, 1962 ▸) or indolo[3,2-*b*]carbazole (Reig *et al.*, 2015 ▸), which is a less π-extended analogue of **1**, arrange in a herringbone packing characterized by edge-to-face C—H⋯π inter­actions. Despite their excellence in organic electronics, the herringbone motif appears as non-optimal for charge transport (Campbell *et al.*, 2017 ▸) because of the generally smaller direct π–π orbital overlap (Yao *et al.*, 2018 ▸). Consequently, the elongation of the aromatic system from indolo[3,2-*b*]carbazole to diindolocarbazole seemingly results in a favourable, more flattened, γ disposition. From a structural point of view, these results support the potential of com­pound **1** as a semiconductor for OTFT devices.

Another inter­esting point relates to the role of the alkyl chains, which frequently determine the inter­molecular arrange­ment. In fact, alkyl­ation is often found to suppress the herringbone packing in favour of a more π-stacked one (Klues & Witte, 2018 ▸). Even in the above-mentioned example of indolo[3,2-*b*]carbazole, the variation of the *N*-alkyl­ation pattern is able to modulate and shift the inter­molecular packing of the core (Zhao *et al.*, 2012 ▸). In the case of **1**, the presence of C—H⋯π inter­actions is detected between the alkyl chains and the aromatic systems of contiguous diindolocarbazole mol­ecules along the *c* axis. Specifically, all three methyl­ene groups adjacent to the N atoms provide C—H⋯π inter­actions, thus reinforcing the packing in this direction.

**Figure 11:**
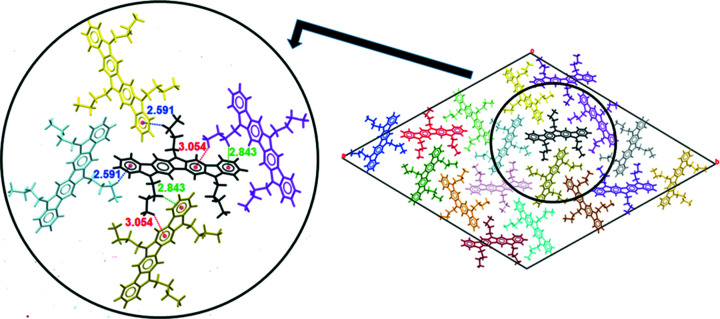
C—H⋯π inter­actions (H⋯centroid distances in Å) observed among various mol­ecules of **1** in the (001) plane.

On the other hand, additional C—H⋯π inter­actions are also formed between mol­ecules belonging to the same plane. In this case, the inter­actions involve two of the three butyl chains: specifically, two methyl­ene and one terminal methyl fragment establish inter­molecular inter­actions with the aro­matic rings of surrounding mol­ecules (Fig. 11 ▸). Thus, the alkyl­­ation of all three N atoms increases the number of inter­actions in all directions and strengthens the crystal packing. As a whole, it can be concluded that this packing satisfies the required aromatic inter­actions particularly well for the subsequent application of **1** in optoelectronic devices.

## 4. Conclusions

Compound **1** has a ladder-type construction based on the diindolocarbazole core, which features a highly π-conjugated system and three butyl chains bonded to the nitro­gen heteroatoms. Its crystal structure, which was inaccessible through conventional solution methods, has been finally determined from PXRD data. The structural solution proved to be a challenging process, mainly due to the large-volume cell range needed to find the proper trigonal cell, *i.e.* the limiting step in the SDPD process. The space group *R* 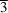 afforded the best fit to the experimental PXRD data, as determined using the Global Optimization Simulated Annealing approach integrated in *TOPAS*. Finally, Rietveld refinement provided a good agreement with an *R*_wp_ value of 4.82%. The analysis of the crystal structure of **1** shows that this linear coplanar mol­ecular structure adopts an inter­esting π-stacked arrangement along the *c* axis with a small π–π stacking distance as close as 3.30 Å, anti­cipating great potential with respect to organic electronics. The presence of the three *N*-alkyl chains also increases the number of inter­molecular inter­actions in all directions, strengthening the packing. Overall, the structural design of **1** leads to a very appropriate mol­ecular arrangement that should grant an effective charge transport. Therefore, the elucidation of organic crystal structures provides crucial information regarding the potential of such materials. In this way, the solution *via* PXRD is confirmed as a helpful alternative towards the understanding, evaluation and eventual application of materials of inter­est in organic electronics in which conventional SXRD is not available.

## Supplementary Material

Crystal structure: contains datablock(s) global, I. DOI: 10.1107/S2052520622001858/aw5063sup1.cif

Structure factors: contains datablock(s) mostra_B. DOI: 10.1107/S2052520622001858/aw5063Isup2.hkl

CCDC reference: 2115611

## Figures and Tables

**Table 1:** Structure solution for the possible space groups

Space group	Multiplicity	*Z*/*Z*′	*R*_wp_	Chemical sense
*R*3	9	18/2	47.9	No
*R* 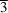	18	18/1	15.4	Yes
*R*32	18	18/1	44.9	No
*R*3*m*	18	18/1	37.7	No
*R*3*c*	18	18/1	37.9	No
*R* 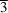 *m*	36	18/0.5	37.4	No
*R* 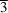 *c*	36	18/0.5	34.2	No

**Table 2:** Crystal data and structure refinement parameters of **1**

Empirical formula	C_36_H_39_N_3_
Formula weight	513.71
Temperature (K)	298
Wavelength (Å)	1.54180
Crystal system	Trigonal
Space group	*R* 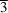
*a*, *b*, *c* (Å)	52.8790 (14), 52.8790 (14), 5.36308 (13)
Volume (Å^3^)	12987.1 (8)
*Z*, *Z*′	18, 1
Density (calc.) (Mg m^−3^)	1.182
Measured 2θ range	2.007 to 69.9840
Stepsize (°)	0.013
Measured data points	5230
	
**Rietveld refinement details**	
Profile function	Double-Voigt
2θ range used (°)	3.2 to 69.98
No. reflections	1257
Data points	5138
Parameters	62
*R*_wp_ (%)	4.82
*R*_p_ (%)	3.54
*R*_Bragg_ (%)	2.11
GoF	4.05
CCDC deposition No.	2115611
